# Salvage of Hindfoot Charcot with Osteomyelitis and Ulceration: A Case Report

**DOI:** 10.3390/medicines9120061

**Published:** 2022-12-02

**Authors:** Khalid Hasan, Sreenivasulu Metikala, Madana Mohana R. Vallem

**Affiliations:** Department of Orthopedics, Virginia Commonwealth University (VCU), Richmond, VA 23298, USA

**Keywords:** Charcot arthropathy, ulceration, osteomyelitis, salvage, internal fixation, staged

## Abstract

Diabetic Charcot arthropathy of the ankle, due to the presence of multiplanar deformities, and associated medical comorbidities, poses a challenge for treating physicians. The situation becomes more complicated when accompanied by ulceration and osteomyelitis, leaving limited salvage options. We present a case of advanced Charcot ankle arthropathy with osteomyelitis and ulcerated hindfoot. It was managed by talectomy and antibiotic-impregnated cement beads, followed by hindfoot arthrodesis using a retrograde intramedullary nail six weeks later. This two-stage reconstruction approach resulted in an ulcer-free, stable, plantigrade foot at one-year postoperative follow-up.

## 1. Introduction

It is a challenging task to treat Charcot neuroarthropathy (CN) of the ankle and hindfoot, even for experienced orthopedic surgeons [1]. Individuals with CN are often not the healthiest patients either. Many of them suffer from peripheral vascular disease, diabetes mellitus, renal failure, and rheumatoid arthritis, among other medical comorbid conditions [2]. Both external injury from trauma and internal pressure from exostosis lead to foot ulceration, followed by recurrent infections [3]. Commonly, a Charcot foot ulcer goes unrecognized due to polyneuropathy and is therefore recognized late in the disease process when osteomyelitis has already set in [4]. A multidisciplinary team approach is necessary to salvage this combination of Charcot deformity accompanied by deep-seated foot ulceration with osteomyelitis.

Surgical debridement with infection control is still the standard of care. Regarding the fixation technique, external fixation using a ring fixator has been a traditional technique [5]. However, the common problems of this method include pin tract infections, often complicated by osteomyelitis and prolonged duration of treatment, resulting in stiffness, osteopenia, and chronic psychological burden [6]. We report a case study of Charcot arthropathy and hindfoot ulceration with exposed and infected bone salvaged entirely by internal fixation using a two-stage reconstruction.

## 2. Case Presentation

**Figure 1:**
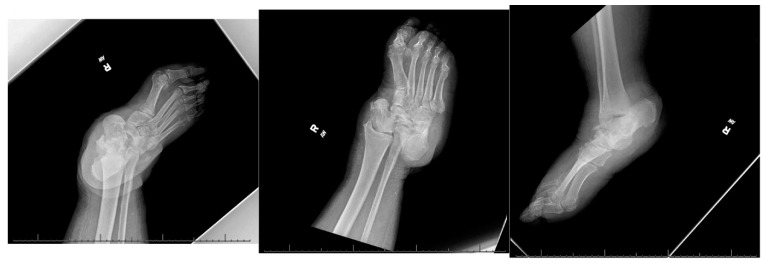
Preoperative radiographic images.

A 66-year-old morbidly obese African American female with a past medical history significant for congestive heart failure (CHF), uncontrolled type 2 diabetes mellitus (DM) with peripheral neuropathy, chronic stage 4 kidney disease (CKD), hyperlipidemia, hypertension (HTN), gastroesophageal reflux disease (GERD), obstructive sleep apnea (OSA), multiple joint arthritis, depression, and asthma, among other diagnoses, had presented to the emergency room with right foot pain and swelling for about two weeks. She was diagnosed with cellulitis of the foot and was sent to medicine service for management. The plain radiographs (Figure 1) showed talonavicular dislocation and marked degenerative changes consistent with Charcot arthropathy. She received intravenous (IV) antibiotics for three days and was discharged on oral antibiotics with instructions to follow up in orthopedic clinic. She returned to the emergency department two weeks later with fever and worsening symptoms of foot pain, accompanied by a draining medial hindfoot ulcer.

Infectious work-up was initiated in the emergency department. Her temperature was recorded as 99.6 °F. The routine hematology lab tests showed elevated white cell count of 13.4 × 10^9^/L with 80% neutrophils, low hemoglobin (Hb) of 8.3 g/dL, and positive blood cultures demonstrating staphylococcus aureus and staphylococcus epidermidis sensitive to Vancomycin. She was admitted under medical service, IV antibiotics were initiated, and orthopedics was consulted. Her most recent HbA1c was 8.1% (normal range 4–6%).

Upon clinical examination, the talar head was found to be protruding through a 7 × 15 cm medial hindfoot ulcer. There was edema of the entire lower leg with cellulitis. After a lengthy discussion to decide between salvage reconstruction and primary below-the-knee amputation, a decision was made for a two-stage reconstruction. Stage 1 included radical debridement and local antibiotic delivery via cement beads, followed by stage 2 for reconstruction and internal fixation.

**Figure 2:**
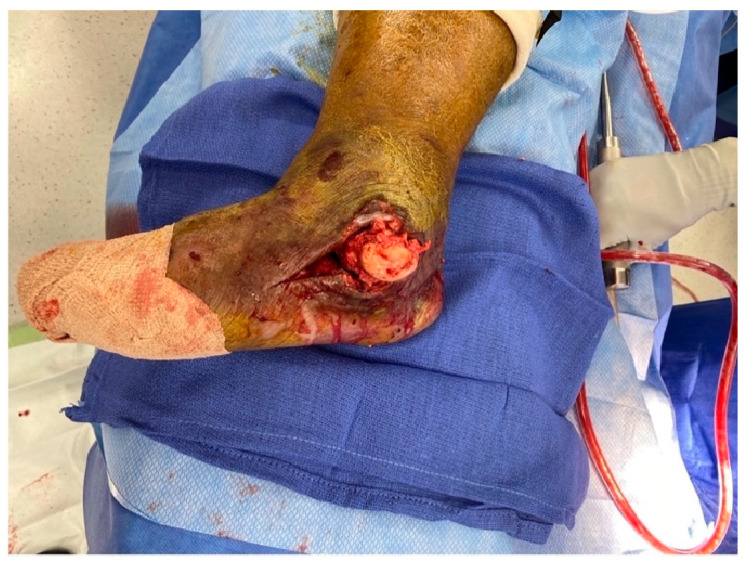
Medial incision.

After adequate medical optimization, the patient was taken to the operating room for the first stage of the procedure. She was placed supine on the operating room table and general anesthesia was induced. After regular sterile prepping, draping and verification of time out, the medial ulcerated wound was extended proximally and distally for adequate exposure (Figure 2).

**Figure 3:**
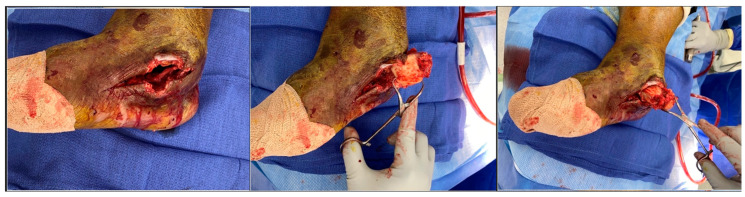
Talus extraction.

The exposed talar head and neck felt soft and mushy, indicating long-standing osteomyelitis changes. Therefore, the decision was taken for a total talectomy. The talar head was grasped using a towel clip, and the talus was excised by meticulous resection of all its soft tissue attachments (Figure 3).

**Figure 4:**
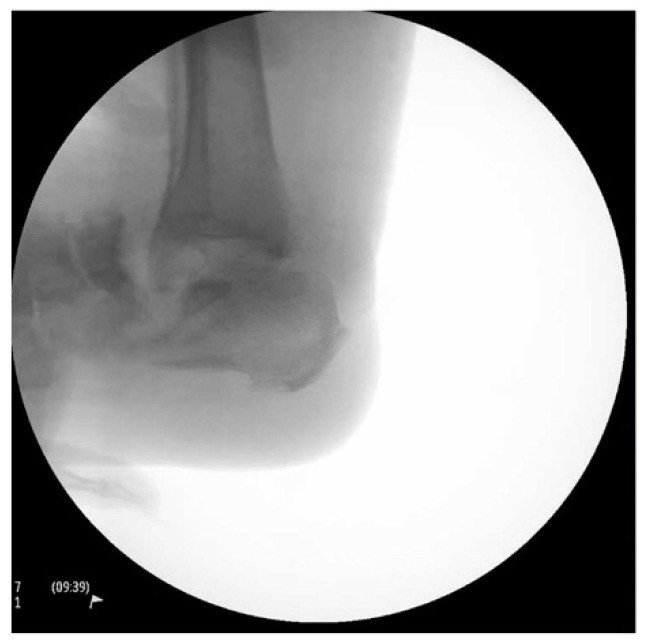
Intraoperative radiograph.

**Figure 5:**
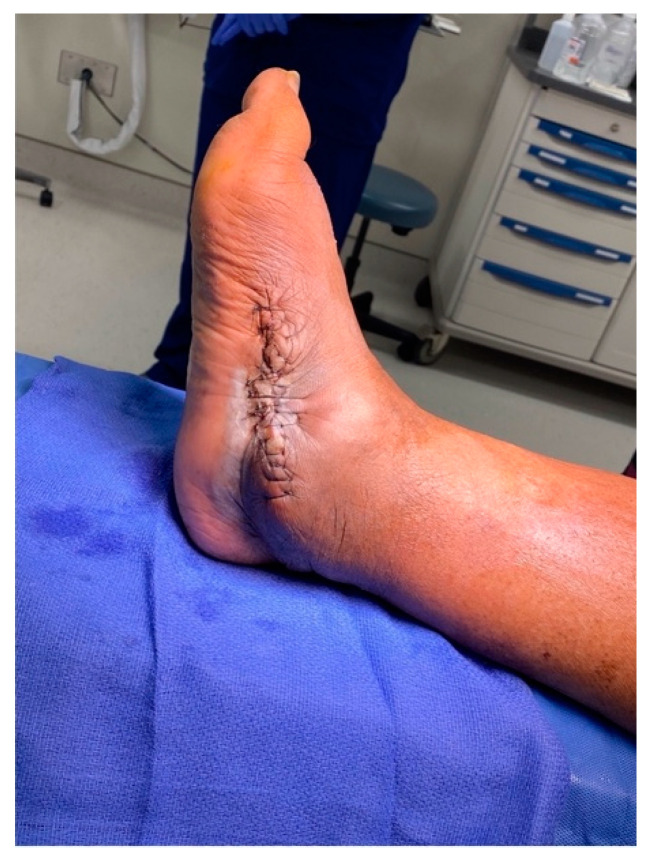
Final wound closure.

**Figure 6:**
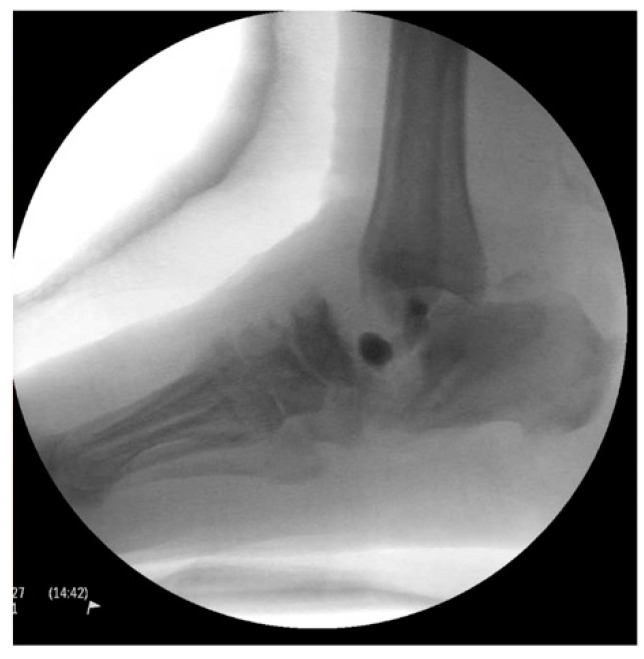
Postoperative image in the cast.

Intraoperative fluoroscopic images confirmed a complete talectomy (Figure 4). This was followed by extensive soft tissue debridement, and a thorough wound lavage. Bone and soft tissue specimens were collected for cultures. The resultant cavity was packed with antibiotic-impregnated cement beads composed of Tobramycin molded on a polydioxanone (PDS) suture. The wound was closed using a nonabsorbable monofilament suture (Figure 5). Sterile dressings were placed, followed by application of a well-padded short-leg posterior plaster splint, which was changed to a cast later (Figure 6).

The patient was advised strict non-weightbearing on the operated extremity. The intraoperative cultures grew Staphylococcus aureus. The patient was sent home with a six-week course of culture-specific antibiotic therapy via a peripherally inserted central catheter (PICC) line as per the recommendations of the infectious disease (ID) team.

**Figure 7:**
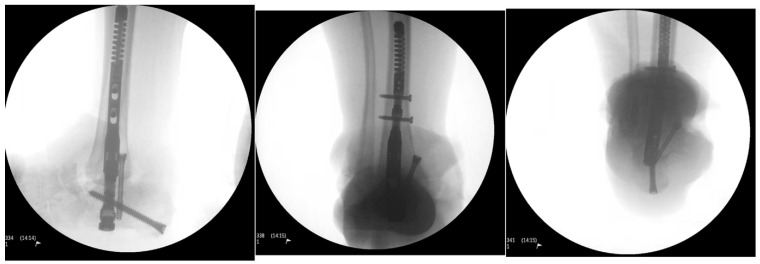
Intraoperative fluoroscopic images showing restoration of axial alignment with retrograde hindfoot nail and headless compression screw.

The surgical wound healed primarily, and the patient completed the six-week parenteral antibiotic course without any incidents. The patient remained afebrile, and the infection blood markers returned to normal range. At eight weeks, the second stage of surgical reconstruction was planned as an outpatient surgery. Given the patient’s comorbidities and less-than-desirable living conditions in a rural setting, internal fixation was preferred over external fixation. A retrograde hindfoot intramedullary nail was selected for internal fixation. The former medial skin incision was reopened, removing the cement beads. The joint surfaces of the tibia, calcaneus and the navicular bones were prepared using a combination of rongeurs, curettes, and drills to obtain raw surfaces. A separate lateral incision over the distal fibula was created, approaching the distal fibula. After meticulous subperiosteal dissection, a 3 cm distal fibula was resected and nibbled into cancellous bone chips. Next, the calcaneus was manually reduced, aligning the posterior tuberosity with the distal tibia and anterior process with the navicular bone. The calcaneus was fashioned minimally to facilitate adequate reduction using rongeur and rasp. Once the reduction was deemed acceptable by biplanar fluoroscopy, multiple Kirshner wires were inserted for provisional fixation. A 10 mm intramedullary hindfoot nail was inserted in a retrograde fashion using standard technique and stabilized with one calcaneal and two tibial locking screws. A headless compression screw was inserted in an antegrade fashion outside the internal nail to supplement the fixation of the tibio-calcaneal interface (Figure 7).

**Figure 8:**
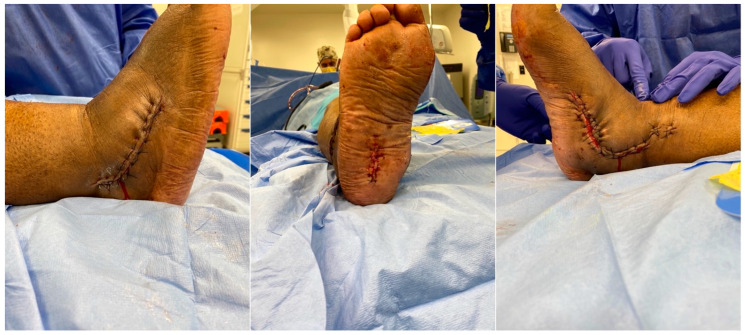
Intraoperative clinical images after fixation.

A percutaneous Achilles lengthening was performed. All surgical wounds were irrigated with normal saline and closed in layers, obtaining a tension-free approximation of skin edges (Figure 8). The patient was placed in a well-padded posterior plaster splint, and discharged home with non-weight-bearing instructions.

**Figure 9:**
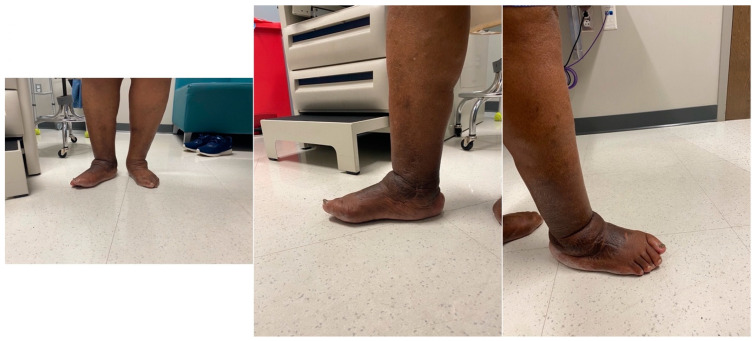
The final one-year post-operative clinical images.

**Figure 10:**
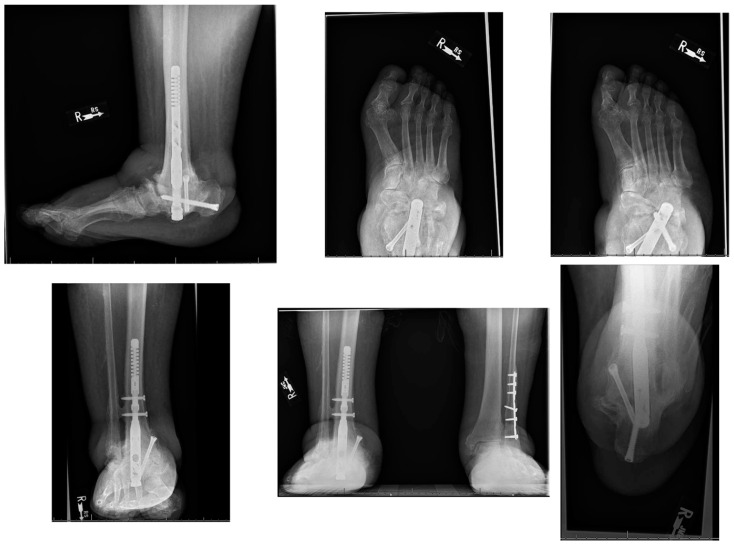
The final one-year postoperative weightbearing radiographs.

The sutures were removed at three weeks, confirming primary healing of all surgical incisions. The patient transitioned into a fiberglass cast, allowing protected weight bearing six weeks after surgery. At three months, a walking boot was provided for further protection, which she discontinued due to poor fitting. She was then advised to use diabetic shoe wear with a heel lift to address the 1.5 cm shortening of the operative extremity. The patient gradually returned to previous activities, fully weightbearing on the operative extremity. At the final one-year postoperative follow-up, patient had no pain or ulcerations in the foot (Figure 9). The final weightbearing radiographs demonstrated adequate osseous fusion with the stable position of the hardware (Figure 10). She was walking in her regular diabetic shoes with stable gait and plantigrade foot.

## 3. Discussion

Salvage of Charcot neuroarthropathy complicated by a hindfoot ulcer and osteomyelitis is a complex situation [7]. It frequently requires multiple surgeries, long courses of antibiotics, and prolonged immobilization without any assurance of complete eradication of infection. It is not uncommon to elect for primary amputation considering the morbidity of the protracted course of reconstructive treatment. In this case report, we described a two-stage successful reconstruction with a multidisciplinary approach, including internal medicine, orthopedic, and ID team physicians, resulting in a pain-free, stable plantigrade foot with intact skin and soft tissues.

Several surgical procedures have been described with varying results. The history of talectomy in foot surgery dates back to the sixteenth century and has been performed for multiple reasons in both adult and pediatric patients [8]. Tibio-calcaneal arthrodesis was first described in 1943 for talar avascular necrosis secondary to a fracture–dislocation [9].

The aim of surgical intervention in an infected Charcot foot with ulceration is to eradicate the infection and obtain a stable, plantigrade foot that will allow the patient to ambulate with or without orthoses without causing any future ulcerations [10]. Satisfactory results have been shown with a single-stage procedure using a preconstructed static circular external fixator [11]. Antibiotic-coated nails have been reported in a limited number of patients who failed the external fixator treatment [12]. A combination of talectomy and tibio-calcaneal arthrodesis was described for a Charcot foot deformity, but internal fixation was reserved for cases without foot ulcers and osteomyelitis [13,14]. Either external fixation or a hybrid fixation technique is favored in infected situations [15]. However, external fixators, in general, are known for several practical problems with psychological impacts, primarily due to lengthy periods, with an average of six months, of retaining the fixator device [16].

Besides a multidisciplinary approach, several technical aspects contributed to a successful salvage in this situation. The debridement during the first stage was extensive, without leaving any infected and devitalized tissues behind in anticipation of the later reconstruction. The goal was to radically excise all infected tissues, including bone, and obtain a primary tension-free wound closure. For the same reason, a bone cement block was avoided in the dead space created after talectomy, which frequently precludes a tension-free approximation of skin edges. Instead, we placed antibiotic-impregnated cement beads, which were smaller in size and allowed primary closure, as well as functioning as a carrier for the local delivery of antibiotics. Additionally, a two-week antibiotic holiday was critical before the second surgery to evaluate for residual infection by repeating the serological infection markers, which were within normal range.

During the second stage, the dual incision (medial and lateral) approach was made to avoid excessive intraoperative retraction and stretching of the skin and soft tissue, once again allowing primary closure with no tension of the sutured margins.

The rarity of our case study lies in implementing the internal fixation method for a hindfoot Charcot complicated by a deep ulcer with exposed talus and osteomyelitis. A staged approach facilitated adequate control of deep infection before the definitive internal fixation, salvaging this complex situation. To our best knowledge, this approach has not been reported yet in a similar clinical setting.

## 4. Conclusions

Charcot neuroarthropathy of hindfoot associated with plantar ulceration and osteomyelitis is an arduous situation. It necessitates a multidisciplinary approach to minimize the risk of amputation. Although an external fixator is a traditional method, internal fixation may be considered in selected patients. A two-stage approach provides a better environment for adequate infection control for successful salvage.
